# EXTREME HEAT VULNERABILITY AMONG OLDER ADULTS: A MULTILEVEL RISK INDEX FOR PORTLAND, OREGON

**DOI:** 10.1093/geroni/igad104.1003

**Published:** 2023-12-21

**Authors:** Jacklyn Kohon, Katsuya Tanaka, Dani Himes, Eiji Toda, Paula Carder, Bryant Carlson

**Affiliations:** Portland State University, Portland, Oregon, United States; Shiga University, Hikone, Shiga, Japan; Institute on Aging, Portland, Oregon, United States; Portland State University, Portland, Oregon, United States; Portland State University Institute on Aging, Portland, Oregon, United States; Portland State University, Portland, Oregon, United States

## Abstract

Extreme heat is an environmental health equity concern disproportionately impacting low- income older adults and people of color. Exposure factors, such as living in rental housing and lack of air conditioning, and sensitivity factors, such as chronic disease and social isolation, increase mortality risk among older adults. Older persons face multiple barriers to adaptive heat mitigation, particularly for those living in historically temperate climates. This study measures two heat vulnerability indices to identify areas and individuals most vulnerable to extreme heat and discusses opportunities to mitigate vulnerability among older adults. We constructed two heat vulnerability indices for the Portland, Oregon metropolitan area: one using area scale proxy measures extracted from existing regional data and another at the individual scale using survey data collected following the 2021 Pacific Northwest Heat Dome event. These indices were analyzed using principal component analysis (PCA) and Geographic Information Systems (GIS). Results indicate that the spatial distribution of areas and individuals vulnerable to extreme heat are quite different. The only area found among the most vulnerable on both indices has the largest agglomeration of age- and income-restricted rental housing in the metropolitan area. Due to spatial variations in heat-related risk at the individual and area scales, measures addressing heat risk should not be spatially uniform. By focusing resources on older adult individuals and areas in particular need of assistance, heat risk management policies can be both highly efficient and cost-effective.

